# Geropotency: Increased malignant potential of aging neural progenitors

**DOI:** 10.18632/aging.100514

**Published:** 2012-12-12

**Authors:** Andrei M. Mikheev, Elizabeth A. Stoll, Rohan Ramakrishna, Svetlana A. Mikheeva, Philip J. Horner, Robert C. Rostomily

**Affiliations:** Department of Neurological Surgery, institute for Stem Cell and Regenerative Medicine, University of Washington School of Medicine, Seattle, WA 98195

In human adult gliomas increased patient age is a robust predictor of malignant clinical behavior evidenced by increased incidence, histologic grade, resistance to therapy and shortened patient survival [1]. Despite these associations, the mechanisms responsible for age-related increases in glioma malignancy are poorly understood; a deficiency compounded by the lack of informative animal models. Neural stem/progenitor (NSPCs) cells are the presumed cells of glioma origin. Using a syngeneic mouse glioma model we showed that the “age” of neural stem and progenitor cells (i.e., the age of donors from which NSPCs were isolated prior to transformation) dictated survival in same-aged hosts and differently-aged (3mo and 20mo) hosts [1]. Surprisingly, host age had no significant effect on survival [1]. After malignant transformation, aging in NSPCs translated to increased invasive potential, genomic instability, resistance to genotoxic stress (radiation and alkylating chemotherapy) and tolerance of hypoxia.

These observations suggest that aging related changes in putative glioma cells of origin contribute to increased malignant potential. However, many aging associated changes observed in normal NSPCs would seem more likely to inhibit malignant potential. For example, with aging there is an overall attrition of NSPCs associated with acquisition of a senescent phenotype with decreased overall proliferation and self-renewal [2]. How then does a senescing cell population generate a more robust cancer cell? Emerging understanding of aging NSPCs suggests that mechanisms associated with aging may actually prime NSPCs for enhanced malignant potential. Conceivably, the increased genomic instability and tolerance of hypoxic stress with aging could enhance NSPC “malignant fitness” or potential [1]. In addition, although less proliferative overall, a small sub-population of aging NSPCs display an increased propensity to re-enter the cell cycle and by conferring a selection advantage could also enhance their malignant potential [3]. Presumably, after malignant transformation the age-dependent state of malignant “pre-conditioning” present in normal NSPCs translates into differential growth advantages within the brain and glioma micro-environment. Genomic instability and hypoxia are speculated to be among the mechanisms fundamental for defining malignant potential and its manifestations of increased invasion, proliferation and treatment resistance. Genomic instability could drive emergence and selection of hypoxia tolerant and responsive cells in the aging brain microenvironment while activation of hypoxic responses in turn could foster acquisition of or enhancement of genomic instability.

Finally, senescent and malignant states share common features of altered chromatin structure, epigenetic changes and DNA damage. Therefore, age-dependent mechanisms that regulate DNA integrity and DNA damage responses may provide clues to reconciling the co-existence of senescence and enhanced malignant potential. One candidate mechanism could be inverse changes observed in p53 and p16 tumor suppressor (TS) proteins in normal NSPCs with aging [1, 2]. In young (3mo) NSPCs p16 is not detectable while robust levels of p53 basal and inducible expression and target gene activation (p21) are present. Conversely, in aged (18mo) NSPCs, p16 expression is dramatically increased concurrent with a dramatic abrogation of p53 activity [4]. Increased p16 contributes to age-related attrition of NSPCs in the SVZ through effects on proliferation and self-renewal [2] but its functional relevance for age-acquired malignant potential is not known. Conversely, abrogation of p53 activity with age could promote increased malignant potential through its known pleiotropic functions to regulate cell cycle progression, apoptosis/senescence, genomic integrity, glycolysis, HIF1 mediated hypoxic responses and invasion. Therefore, differential expression and function of the p16/p53 tumor suppressors, possibly in response to age-related accumulation of DNA damage, represents a candidate mechanism underlying the concurrent acquisition of increased senescent and malignant potential in aging NSPCs. An additional mechanism related to p53 that may also contribute to age-dependent NSPC malignant potential is activation of the mTOR pathway in aging NSPCs (unpublished observation). Decreased p53 may contribute to mTOR activation [5] which in turn may prime aged NSPCs for increased malignant potential. Of interest, mTOR also has been linked to epithelial stem cell senescence [6,7]; therefore, persistence of increased mTOR activity in a sub-set of aged NSPCs that evade senescence may ultimately provide a growth advantage upon malignant transformation.

**Figure 1:**
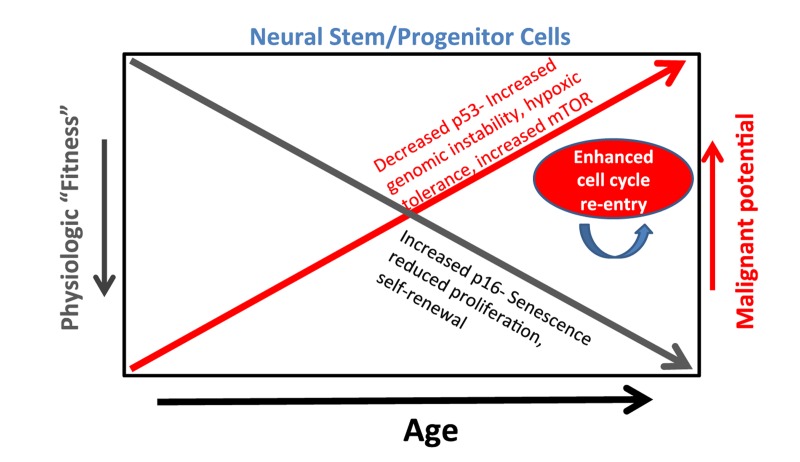
A model whereby mechanisms of aging concurrently contribute to reduced physiologic “fitness” and increased malignant potential of neural stem/progenitor cells. Reduced NSPC “fitness” regulated in part by increased p16 manifests as increased NSPC senescence, reduced proliferation and self-renewal. Conversely, increased NSPC malignant potential or “preconditioning” regulated by decreased p53 function contributes to increased genomic instability, hypoxic tolerance, and possibly mTOR activity. Finally, the rapid cell cycle re-entry [3] and presumed selection advantage of a sub-population of aging NSPCs provides another potential mechanism by which aging NSPCs acquire increased malignant potential.

The incorporation of cell-intrinsic and micro-environmental influences of aging into a glioma model was anticipated to reveal age-related mechanisms relevant to human glioma malignancy. In two respects- increased resistance to genotoxic agents and increased activation of HIF-1 hypoxia response genes- this model recapitulated features associated with age-dependent malignancy of human gliomas. Aging human glioma cells/tumors are more resistant to genotoxic stresses (radiation and alkylating chemotherapy) [1, 8] but in line with age-related increases in VEGF expression, they are more responsive to the VEGF inhibitor bevacizumab [9]. This latter observation echoes the increased activation of hypoxia response genes (HRGs) including VEGF seen in aged transformed NSPCs as well as a general increase in HRG expression in aged human glioma patients [1]. Incorporating aging in an animal glioma model therefore served to identify shared mechanisms that contribute to normal aging and acquisition of malignant potential in NSPCs (Figure 1). Further, the preliminary results provide proof of principle that accounting for aging has relevance in the pre-clinical setting.
